# Fluorescent Beads Are a Versatile Tool for Staging *Caenorhabditis elegans* in Different Life Histories

**DOI:** 10.1534/g3.116.030163

**Published:** 2016-04-29

**Authors:** Liberta Nika, Taylor Gibson, Rebecca Konkus, Xantha Karp

**Affiliations:** Department of Biology, Central Michigan University, Mount Pleasant, Michigan 48859

**Keywords:** fluorescent beads, molting, developmental stage, dauer, *C. elegans*

## Abstract

Precise staging of *Caenorhabditis elegans* is essential for developmental studies in different environmental conditions. In favorable conditions, larvae develop continuously through four larval stages separated by molting periods. Distinguishing molting from intermolt larvae has been achieved using transgenes with molting reporters, therefore requiring strain constructions, or careful observation of individuals for pharyngeal pumping or behavioral quiescence. In unfavorable conditions, larvae can enter the stress-resistant and developmentally arrested dauer larva stage. Identifying dauer larvae has been based on their ability to withstand detergent selection, precluding identification of recovering animals or of mutants with defects in dauer morphogenesis. Here, we describe a simple method to distinguish molting larvae or dauer larvae from intermolt larvae that bypasses the limitations of current methods. Fluorescent latex beads are mixed with the bacterial food source and ingested by intermolt larvae and adults. Molting and dauer larvae do not feed, and therefore lack beads in their digestive tract. The presence of beads can be determined using a dissecting microscope at magnifications as low as 100 ×, or by using a wormsorter for high-throughput experiments. We find that continuously developing bead-lacking larvae display hallmarks of molting, including expression of the *mlt-10*::*gfp* molting marker and a lack of pharyngeal pumping. Furthermore, wild-type and mutant dauer larvae produced by any of three common methods are accurately identified by a lack of beads. Importantly, this method is effective in SDS-sensitive mutant backgrounds and can identify recovering dauer larvae, a stage for which there is no other method of positive selection.

*C. elegans* can develop through different life histories depending on environmental conditions. In favorable conditions, *C. elegans* develops rapidly and continuously through four larval stages (L1–L4) punctuated by molts, before reaching adulthood within about 3 d. However, if conditions in the L1 stage are unfavorable for growth, larvae will enter the extended L2d stage, during which they prepare for dauer entry and continue to monitor environmental conditions (Golden and Riddle 1984). After the L2d stage, larvae can either molt to L3 and continue developing, or molt into the developmentally arrested and stress-resistant dauer larva stage (Figure 1A) (Golden and Riddle 1984). At each molt, ecdysis is preceded by a period of lethargus, a quiescent period marked by little to no pharyngeal pumping, during which larvae synthesize new cuticles (Sulston and Horvitz 1977). Hereafters the combined process of lethargus and ecdysis is referred to as “the molt.” Although typical molting periods last 1–2 hr, the L2d-dauer molt lasts approximately 12 hr (Golden and Riddle 1984).

**Figure 1:**
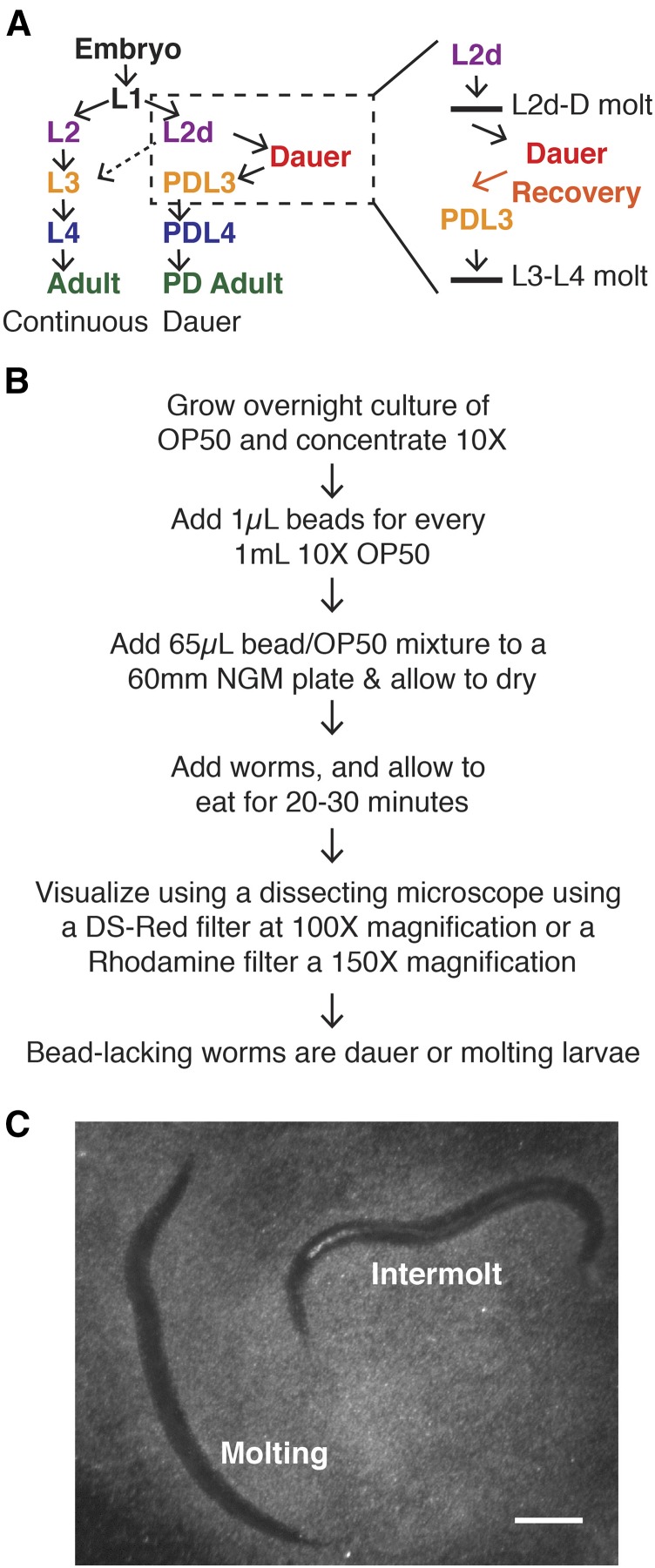
Summary of bead method. (A) Diagram of *C. elegans* life history choice. Continuous development (left) occurs in favorable environmental conditions, whereas dauer development (right) occurs in adverse environmental conditions. The boxed region is shown in more detail on the far right. Horizontal lines represent molts. (B) Summary of the method used in this paper. Lower magnifications are possible for older larvae or adults. (C) Representative images of L4 (intermolt) and L4 molt staged hermaphrodites on bead-containing plates. This image was taken on a Leica MZ16F dissecting microscope with a DS-Red filter using a 1 × objective and 10 × zoom to produce 100 × magnification. Scale bar = 0.1 mm. L, larval stage; OP50, OP50 strain of *E. coli*; PD, postdauer.

Rough staging of *C. elegans* larvae can be carried out using body size and gross morphology visible under the dissecting microscope, whereas precise staging requires either a stage-specific transgenic marker or detailed examination of anatomy. The ability to distinguish molting and intermolt larvae would markedly improve the precision of staging under the dissecting microscope, and would also help to refine the more detailed anatomical observations. Transgenes encoding fluorescent molting markers are useful tools to track molting (Frand *et al.* 2005). A recently published method uses bioluminescence to quantitatively stage *C. elegans* larvae, including distinguishing molting and intermolt larvae, providing an important new tool to the *C. elegans* field (Olmedo *et al.* 2015). However, these methods rely on transgenes that must be crossed into any strains that are to be examined.

In addition to staging larvae during “normal” development in favorable laboratory conditions, staging larvae during the dauer life history is also important for many applications. The pathways that regulate dauer formation also regulate aging, metabolism, stress-resistance, and neurobiology. Thus, a wide range of researchers work with dauer formation mutants, and may need to identify dauer larvae.

Dauer larvae are morphologically, metabolically, behaviorally, and epigenetically distinct from other stages, including their counterpart in continuous development, early L3 stage larvae (Cassada and Russell 1975; O’Riordan and Burnell 1989; Wadsworth and Riddle 1989; Hall *et al.* 2010; Karp *et al.* 2011). Dauer larvae can survive for months without feeding (Klass and Hirsh 1976). If conditions improve, dauer larvae undergo a recovery process lasting several hours, during which they resume feeding and undergo metabolic changes and changes in gene expression (Cassada and Russell 1975; Reape and Burnell 1990; Houthoofd *et al.* 2002; Wang and Kim 2003). After recovery, dauer larvae resume development through postdauer L3 (PDL3) and PDL4 stages that are developmentally equivalent to continuously developing L3 and L4 staged larvae (Liu and Ambros 1991; Euling and Ambros 1996; Braendle and Félix 2008; Karp and Greenwald 2013). Notably, there is no molt between dauer and postdauer L3, making these stages difficult to distinguish (Cassada and Russell 1975) (Figure 1A).

The most common method used to isolate dauer larvae is resistance to 1% SDS (sodium dodecyl sulfate) (Cassada and Russell 1975). SDS resistance is based on two dauer-specific characteristics. First, the dauer cuticle is thick and particularly resistant to environmental insult. Second, dauer larvae do not ingest the detergent solution due to the presence of a buccal plug sealing the mouth. Additionally, lack of pumping provides some protection from ingestion even in the absence of the plug (Cassada and Russell 1975). This method works well to isolate wild-type dauer larvae from mixed populations; however, it is less useful when working with mutants with defective dauer cuticles that impair SDS resistance. SDS-sensitive mutants include the following gene categories: 1) genes encoding components of the dauer cuticle (Sebastiano *et al.* 1991; Sapio *et al.* 2005), 2) genes that regulate the fate of cuticle-secreting hypodermal cells, such as heterochronic genes (Liu and Ambros 1989; Karp and Ambros 2012), and 3) certain genes involved in the regulation of dauer formation (Albert and Riddle 1988; Gottlieb and Ruvkun 1994; Antebi *et al.* 1998). The latter category may be considered dauer-like or partial dauer larvae (Albert and Riddle 1988).

Another limitation of SDS resistance arises when trying to identify or quantify nondauer larvae from a population of dauer larvae. In this case, application of SDS kills the desired population. For example, recovering dauer larvae (or PDL3 larvae) appear similar to dauer larvae but are sensitive to SDS. Populations of wild-type recovering dauer larvae can be obtained by SDS selecting dauer larvae, followed by incubation in favorable conditions for several hours (Cassada and Russell 1975). However, some strains do not recover synchronously, making chronological time less effective as a criterion. These strains include “Daf” mutants that are defective in the regulation of dauer formation (Vowels and Thomas 1992; Thomas *et al.* 1993; Vowels and Thomas 1994; Malone *et al.* 1996). Having a tool to identify individual recovering dauer larvae would enable the study of the process of recovery and the ability to perform genetic screens focused on this stage. Other examples of experiments where the nondauer population is important include genetic screens for dauer defective mutants and quantitative studies of dauer formation (*e.g.*, Diaz *et al.* 2014).

Here, we report the use of fluorescent latex beads to distinguish intermolt from molting larvae, and to distinguish dauer larvae from other stages. These beads have been used previously to study various aspects of feeding and defecation (*e.g.*, Fukushige *et al.* 1998; Raizen *et al.* 2012), but have not been used to monitor developmental stage or identify dauer larvae. We validated the use of beads to identify intermolt larvae throughout development, as well as dauer larvae formed by each of three common methods for inducing dauer formation. Lack of beads correctly identifies molting larvae and dauer larvae, including SDS-sensitive mutants, whereas the presence of beads correctly identifies intermolt and recovering dauer larvae.

## Materials and Methods

A summary flowchart of the method used in this paper is shown in Figure 1B. Figure 1C displays a representative image of worms with and without beads, as viewed under a dissecting microscope.

### Nematode strains

The following strains were used in this study. N2: wild-type, CB1370: *daf-2(e1370)*: CB1372, *daf-7(e1372)*, VT2317: *daf-16(mgDf50);* *daf-7(e1372)*, CB1: *dpy-1(e1)*, VT1207: *lin-4(e912);* *mir-48mir-241(nDf51);* *lin-14(n179) mir-84(n4037)*, GR1395: *mgIs49[mlt-10::gfp-pest]* (Frand *et al.* 2005; Hayes *et al.* 2006). All strains were maintained at 20° on Nematode Growth Medium (NGM) plates seeded with the OP50 strain of *Escherichia coli* according to standard procedures (Brenner 1974). Experiments involving dauer formation or continuous development were carried out at 24°, whereas dauer recovery was carried out at 20°.

### Fluorescent beads

Unless otherwise indicated, the beads used were 0.5µm mean particle size (Sigma L3280, red fluorescence). These beads were chosen because they are similar in size to *E. coli*, and they have been successfully used in pharyngeal pumping assays (Fang-Yen *et al.* 2009; Raizen *et al.* 2012). Given that the pharyngeal lumen of L1 larvae is estimated to be 1 µm in diameter (Avery and Shtonda 2003), we did not attempt beads larger than 0.5 µm.

A 5 ml overnight culture of OP50 was centrifuged at 4000 rpm for 7 min. The pellet was resuspended in 0.5 ml LB to concentrate the bacteria 10 ×. For every 1 ml of concentrated OP50, 1 µl of fluorescent beads was added to obtain a 1:1000 ratio (v/v) of beads to bacteria. NGM plates (60 mm) were seeded with 65 µl of the beads/OP50 mixture to generate “bead-containing plates.” The bead/OP50 mixture and/or the seeded plates were used immediately or stored at 4° for up to 8 wk before use. For experiments where worms are allowed to eat for only a limited time (*e.g.*, 20–30 min), the bead/OP50 mixture was spread by tilting the plate so as to cover most of the surface of the plate, in order to reduce the likelihood that a worm lacked beads simply because it had not yet encountered food.

### Microscopy

Experiments describing precise staging, dauer alae, radial constriction, or the L2d-dauer molt were carried out on slides on a compound microscope using a 63 × objective and DIC optics (Zeiss AxioImager D2). For these experiments, the presence of beads was also determined using the compound microscope using an Illuminator HXP 200C light source and a HE Cy3 shift free filter. Larvae within the L2d-dauer molt were identified by indistinct dauer alae, together with an extra cuticle (Singh and Sulston 1978).

Experiments comparing beads to pharyngeal pumping or *mlt-10*::*gfp* expression were visualized using a dissecting microscope (Zeiss Stereo V12 fitted with M2 Bio for fluorescence) with an X-Cite 120Q light source, a Rhodamine filter (Kramer Scientific, KSC 296-815D) for red, and a GFP/eGFP filter (Kramer Scientific, KSC 296-833D) for green. This microscope has a maximum zoom of 100 × (10 × eyepieces × 10 × zoom body). To unambiguously visualize beads in the smallest larvae on this microscope, we used either a 10 × objective (Kramer Scientific, KSC 190-975-HR 10x/0.45) or a 1.5 × objective (Zeiss Achromat S 435228-9901) to achieve an overall magnification of 150 ×.

We found that the particular filter set greatly impacts the visibility of the beads. Specifically, a DS-Red filter set (excitation 510–560 nm; emission 590–650 nm) caused the beads to appear brighter, thereby allowing magnification to be reduced markedly compared to the Rhodamine filter (excitation 520–570 nm; emission 605–670 nm). With a DS-Red filter, beads could be unambiguously identified using a 1 × objective and a zoom body setting of 10 for an overall magnification of 100 × (10 × eyepieces × 10 × zoom body × 1 × objective). For comparison using a different microscope, we examined worms on bead-containing plates using a Leica MZ16FA stereomicroscope with a DS-Red filter. Here we could unambiguously distinguish bead-containing from bead-lacking larvae at the youngest stages using a 2 × objective and 4 × zoom body setting, for an overall magnification of 80 × (10 × eyepieces × 4 × zoom body × 2 × objective). There was less ambiguity under these conditions than when visualizing the worms at 80 × using the 1 × objective and (10 × eyepieces × 8 × zoom body × 1 × objective), suggesting that it is the quality of the objective rather than the specific magnification that is most important.

We note that, if larvae are removed from bead-containing plates, beads will be emptied from the digestive tract by defecation. Worms placed on food-containing plates begin emptying of beads after 5 min and complete emptying within 35 min (Supplemental Material, Table S1). Worms placed on food-lacking plates begin emptying after 15 min and do not complete emptying within 1 hr (Table S1). Sodium azide (10 mM) can be used to inhibit emptying (see Wormsorter experiments below).

### Distinguishing molting and intermolt larvae

**Figure 2:**
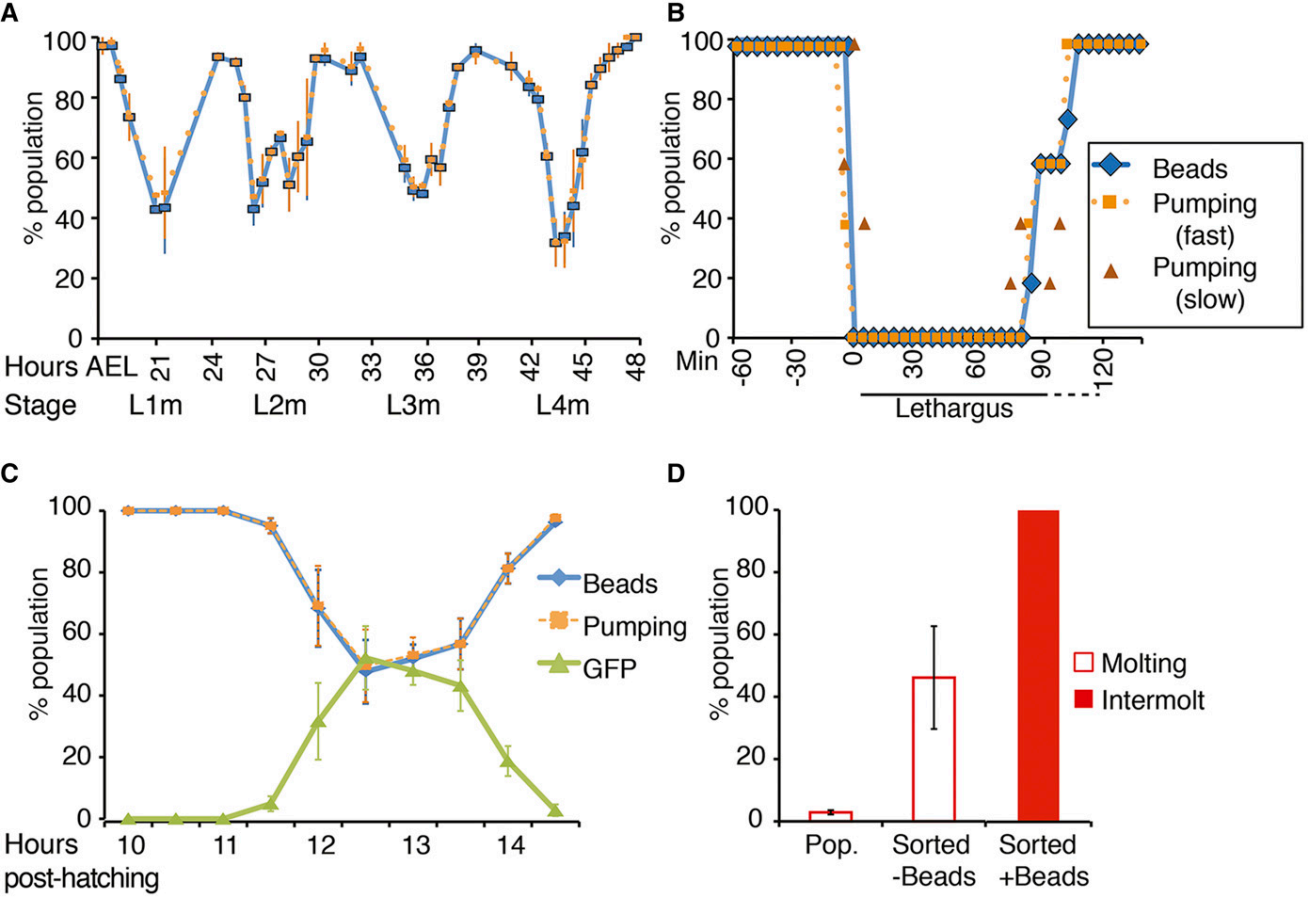
Beads identify intermolt larvae during continuous development. (A) Synchronous populations of N2 larvae grown on bead-containing plates at 24°. The average ± SEM of three independent trials is shown. *n* = 40–83. (B) Individual N2 larvae grown on bead-containing plates at 24° were monitored for beads and pumping every 5 min throughout the L1 molt and the L4 molt. 4–5 independent trials were carried out at each stage consisting of 3–8 larvae per trial. Shown here are the average data from five larvae in one trial at the L1 molt. Details from this trial are shown in Figure S1. The average data from the other trials are shown in Figure S2. Time 0 is defined as the time in min that beads are completely expunged from the digestive tract. (C) A synchronous population of *mgIs49[mlt-10p*::*GFP-pest]* larvae was observed following release from L1 arrest. Around the L1 molt, bead-lacking larvae strongly express GFP (see Figure S3), and fail to pump. The average ± SEM of at least three independent trials is shown. *n* = 71–122. (D) Pop. = % of the unsorted population that is molting, as determined by lack of beads (*n* = 789); Sorted = the animals recovered from the wormsorter (−Beads: *n* = 268 sorted worms; + Beads: *n* = 536 sorted worms). Sorting for animals that lack beads (−Beads) greatly enriches for molting larvae compared to the general population. Sorting for animals that contain beads (+ Beads) produces 100% intermolt or adult animals. Sorted molting larvae lacked beads and pumping whereas sorted intermolt larvae possessed beads and pumped. The average of at least three independent trials ± SEM is shown. Data for all panels were acquired on a dissecting microscope. GFP, green fluorescent protein; L, larval stage.

Synchronous populations of N2 larvae were obtained by letting gravid adult hermaphrodites lay eggs for 1 hr at 24° on bead-containing plates. For population assays, larvae were maintained on a single plate per trial and scored for beads and pumping at the times indicated in Figure 2A. For individual assays (Figure 2B, Figure S1, and Figure S2), approximately 14 hr after egg laying, the now L1-staged larvae were separated on individual bead-containing plates and incubated at 24° for an additional 8 hr. At this point, 3–5 individuals were monitored on the dissecting microscope every 5 min for beads and pumping until the L1 molt was complete.

### mlt-10::gfp-pest

A synchronous population of *mlt-10*::*gfp-pest* animals (GR1395) was obtained by sodium hypochlorite treatment followed by hatching embryos in the absence of food. Embryos were incubated in M9 buffer for 12–16 hr at 24° with gentle agitation. Next, the now synchronous population of L1 larvae was added to bead-containing plates. After an additional 9 hr of incubation at 24°, the animals were scored every 30 min until the L1 molt was completed. The animals were scored for beads, pumping, and GFP using a dissecting microscope as described above. Note that faint GFP was seen in both molting and intermolt larvae with our microscope, but was distinguishable from the strong GFP expression observed during the molt (Figure S3). To limit the background fluorescence, the amount of UV light was reduced by narrowing the aperture diaphragm, and GFP was viewed at 100 × magnification rather than the ≥ 150 × used to view the beads.

### Wormsorter

A COPAS *BIOSORT* (Union Biometrica) platform was employed to sort a large population of continuously developing N2 larvae. A mixed population of N2 hermaphrodites was generated by plating 3–4 L4 staged N2 hermaphrodites on 60 mm bead-containing plates and incubating at 20° for 3–4 d. For these experiments, we used polychromatic red microspheres (Polysciences 19507-5) instead of the red fluorescent beads used above because the latter were poorly visible on the COPAS machine. After 3–4 days of incubation at 20°, there were numerous progeny of various stages and still plenty of food. Animals were washed off of plates with M9, pelleted, and resuspended in 500 µl M9 with 10 mM sodium azide. The sample was incubated at room temperature for 10 min to ensure the animals were anesthetized prior to sorting. Treatment with sodium azide prevented defecation, thereby maintaining any beads within the digestive tract during sorting. M9 was then added to reach a final sample volume of 1 ml that was immediately added to the COPAS sample cup. BIOSORT5292 software was used to operate the platform. The Parameters for Analysis (PMTs) selected were red *vs.* green fluorescence. To identify molting worms, a region containing the lowest possible fluorescence levels for both red and green emissions was selected as the “sort criteria.” Animals displaying levels within the sort criteria were dispensed onto empty NGM plates (containing no food). The collected individuals were visualized using a Zeiss Stereo V12 with M2 Bio dissecting microscope with a DS-Red filter. The animals were given 20 min to recover from sodium azide treatment before scoring individuals for pharyngeal pumping and beads within their digestive tracts. All animals recovered from sodium azide treatment and normal pumping resumed within 10–20 min. However, beads were still present within the digestive tract 60 min after removal from sodium azide.

Note that we do not recommend polychromatic beads for experiments that do not involve the wormsorter for two reasons. First, they are more expensive than the beads from Sigma. Second, we found the polychromatic beads harder to see under the dissecting microscope.

### SDS resistance assay

At least 20 larvae were picked into 100 µl of 1% SDS in a well of a 96-well plate. After 10 min or 40 min of incubation (see Figure S5), the larvae were transferred onto a NGM plates seeded with OP50 (without beads) and incubated at 20°. Larvae were scored either immediately (for 10 min assays) or the next day (for 40 min assays) for survival, as previously described (Cassada and Russell 1975). Animals that responded to mechanical stimuli were considered alive; any other animals were considered dead.

### Isolating dauer larvae from starved plates using fluorescent beads

Approximately five hermaphrodites of a given strain were added to 60 mm NGM plates seeded with OP50 and incubated at 20° for 1–4 wk, or at least several days past the time at which the food supply became exhausted. Worms were washed from a single plate, pelleted, and added to a bead-containing plate. After 30 min, bead-containing and bead-lacking animals were scored for SDS resistance or for dauer alae and radial constriction.

### Pheromone plates

NGM media was prepared without peptone and with streptomycin (50 μg/ml). Two ml of media were added to each 35 mm plate (Golden and Riddle 1984). Crude dauer pheromone was produced by a culture of wild-type (N2) hermaphrodites growing in liquid medium (S Medium) (Stiernagle 2006). Pheromone was extracted from the S buffer by ethanol distillation as previously described (Vowels and Thomas 1994; Zhang *et al.* 2013). 50 µl of crude pheromone was added to each plate. Once dry, plates were seeded with 20 µl of beads + OP50 (1:1000, v/v). Gravid N2 adult hermaphrodites were picked onto a pheromone plate and allowed to lay eggs at 24°. After 2 hr, adults were removed and the progeny developed at 24°.

### Isolating dauer larvae formed by dauer constitutive (Daf-c) alleles

To obtain a semisynchronous population, 20–25 gravid adults were picked onto bead containing plates at 24° for 5 hr, at which point the adults were removed. The beginning of the 5 hr was considered time 0. Developing larvae were observed at the timepoints indicated in Figure 4, A–F. A fluorescence dissecting microscope was used to monitor the presence of beads and pharyngeal pumping, whereas a compound microscope was used to observe beads, dauer alae, radial constriction, and developmental stage.

Because some *daf-16*; *daf-7* larvae grown at the nonpermissive temperature (24°) bypass dauer formation and develop continuously (Larsen *et al.* 1995; Narasimhan *et al.* 2011), and because some *daf-16*; *daf-7* dauer larvae recover spontaneously (Figure S6), beginning at 40 hr after egg laying, some L4 larvae were observed on *daf-16*; *daf-7* plates. After scoring them, these larvae were removed to prevent them from producing progeny that could be confused with the test animals on the plate. The number of larvae bypassing dauer formation varied widely from experiment to experiment, and was not quantified. The percentage of larvae recovering spontaneously is quantified in Figure S6.

### Dauer recovery

#### N2:

Animals were washed off starved plates (from populations grown at 20°) and incubated with 1% SDS with gentle agitation on a nutator for 30 min before pelleting and adding worms to bead-containing plates.

#### Daf-c mutants:

Dauer larvae were obtained as described above. At 50–72 hr after egg laying, bead-lacking dauer larvae were added to bead-containing plates at 20°. Recovering dauer larvae were monitored, and when approximately half of the population displayed beads in their digestive tract (typically ∼24 hr after the shift to 20°) they were tested for SDS resistance.

### Data availability

All strains are available at the *Caenorhabditis* Genetics Center or upon request. Beads are commercially available (Sigma L3280). Detailed experimental data are present in the figures and supplemental material.

## Results

### Using beads to distinguish molting from intermolt larvae

Fluorescent beads have been used to study feeding and defecation (Fukushige *et al.* 1998; Raizen *et al.* 2012), suggesting the possibility that they could be used to differentiate stages that differ in feeding behavior, such as molting and intermolt larvae. Because pumping is essentially suspended during the molt (Cassada and Russell 1975; Byerly *et al.* 1976), we used pumping as a marker of molting. We used a dissecting microscope to monitor pumping in synchronous populations of N2 larvae during development on NGM plates seeded with a 1:1000 (v/v) mixture of red fluorescent beads (Sigma L3280) and OP50 (“bead-containing plates”) (Figure 1C and Figure 2A) (see *Materials and Methods* for details). We found a close correlation between beads and pumping throughout larval development, with a decrease in both beads and pumping during molts (Figure 2A). Developing larvae appear to empty their intestines prior to lethargus, such that nonpumping larvae rarely had beads within their digestive tract (Figure 2A). To more closely examine the correlation between beads and pumping, we followed individual larvae through the L1 and L4 molts (Figure 2B, Figure S1, and Figure S2). We found that larvae in the intermolt period contained many beads throughout their digestive tract and pumped rapidly. In the 5–15 min just prior to lethargus, pumping slowed and larvae emptied their digestive tract of beads. At this time, bead-lacking larvae sometimes exhibited slow pumping. After this initial period, neither beads nor pumping were observed until the end of the molt, when slow pumping resumed a few minutes prior to beads entering the digestive tract. Five minutes after the initial appearance of a few beads in the digestive tract, many beads were again observed in the gut, and pumping was again rapid (Figure 2B, Figure S1, and Figure S2).

In order to follow the molting cycle using a criterion other than pumping, we utilized the *mlt-10*::*gfp-pest* transgene that is expressed in molting larvae (Frand *et al.* 2005). We found that, in continuously developing larvae around the L1 molt, strong expression of *mlt-10*::*gfp-pest* was seen only in bead-lacking larvae (Figure 2C and Figure S3). These results indicate that a lack of beads can be used to isolate molting larvae and, conversely, that the presence of beads can be used to identify larvae in the intermolt period.

Using fluorescence rather than pumping to distinguish molting and intermolt animals may allow large scale experiments where populations are sorted automatically using a COPAS Biosort (“wormsorter”). Indeed, the COPAS device has been shown to be sensitive enough for quantitative measurements of ingested beads in response to the addition of exogenous stimulants or inhibitors of feeding (Boyd *et al.* 2007). As a proof-of-principle, we used a wormsorter to sort out molting or intermolt worms from a mixed stage population. We grew wild-type worms on bead-containing plates to generate a mixed stage population. For these experiments, we used polychromatic beads because the red beads used above were poorly detectable by the wormsorter (see *Materials and Methods* for details). Only 3% of worms in these populations were molting (Figure 2D), which would make manual identification of molting worms quite labor intensive. Using the wormsorter, we found that molting larvae could be enriched 10 × (Figure 2D). Furthermore, intermolt larvae and adults could be sorted away from molting worms with 100% accuracy (Figure 2D). Therefore, beads provide a new transgene-free approach to aid in the identification of molting or nonmolting animals.

### Characterization of bead ingestion during recovery of wild-type dauer larvae

**Figure 3:**
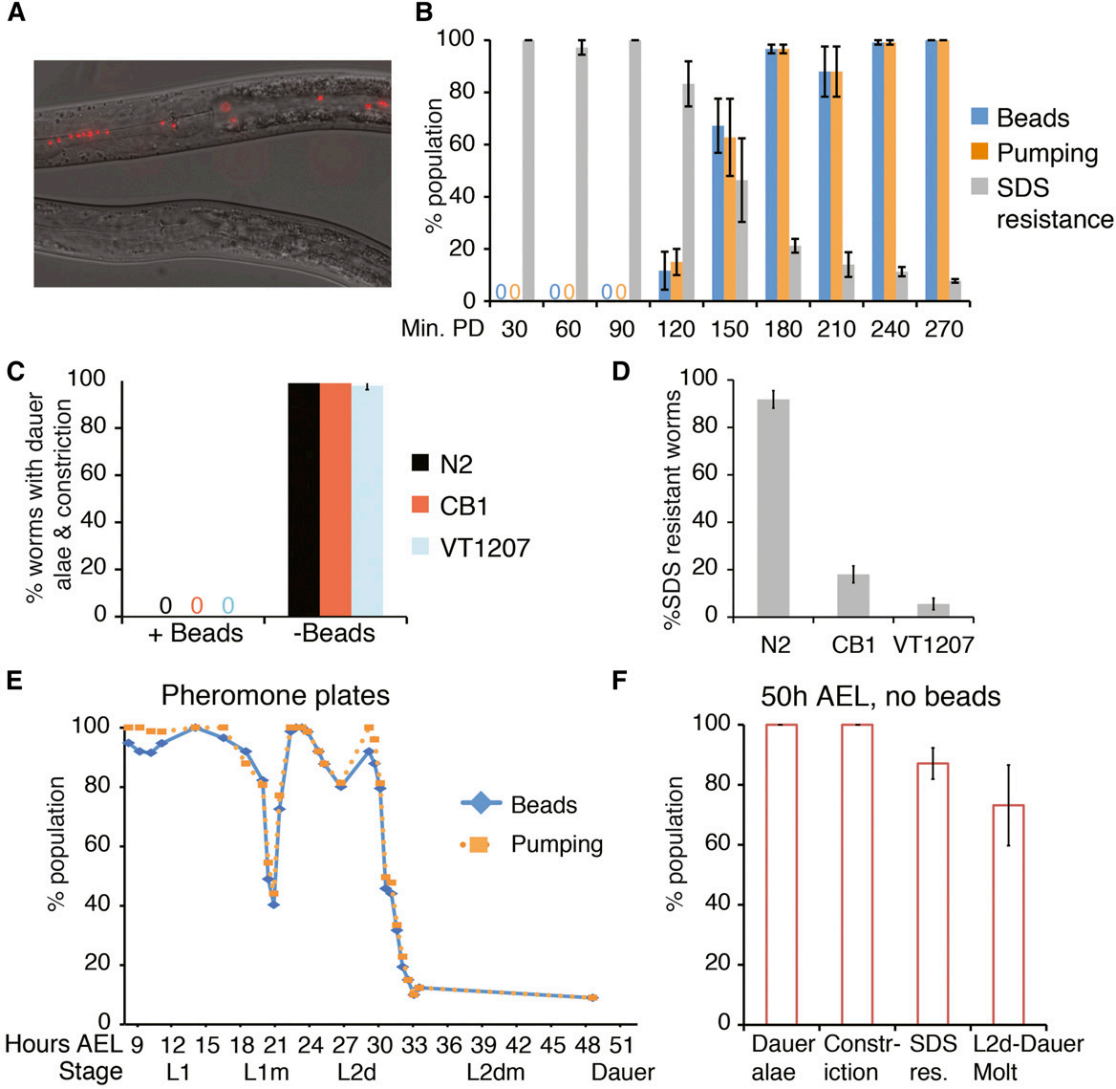
Lack of beads identifies dauer larvae formed in response to starvation or dauer pheromone. (A) Photomicrographs of larvae that have been washed from a starved plate onto a bead-containing plate and allowed to feed for 30 min. Nondauer larvae (top) contain beads in their digestive tracts, whereas dauer larvae (bottom) lack beads. These photos were taken on a compound microscope (63 × objective). (B–D) Averages of at least three independent trials ± SEM are shown. (B) Wild-type SDS-resistant dauer larvae from a starved population were placed on bead-containing plates and allowed to recover at 20° for the indicated times before observing them on a dissecting microscope. Min. PD = Minutes postdauer (*n* > 50). (C–D) Wild-type or mutant animals with abnormal cuticles were washed off starved plates and added to bead-containing plates. N2 = wild-type, CB1 = *dpy-1(e1)*, VT1207 = *lin-4(e912)*; *mir-48 mir-241(nDf51)*; *lin-14(n179) mir-84(n4037)*. After 30 min, they were scored on a compound microscope for dauer alae and radial constriction (*n* > 45) (C), or for resistance to 1% SDS (*n* > 40) (D). (E) Synchronized populations of wild-type larvae were grown at 24° on bead-containing plates supplemented by the addition of crude dauer pheromone. Larvae were monitored on a dissecting microscope before and during dauer formation. Data from multiple independent trials were aggregated and the total numbers are shown here. *n* = 25–83. (F) At 50 hr after egg laying (AEL), bead-lacking larvae from (E) were examined on a compound microscope for the indicated characteristics. Many larvae were still within the L2d-dauer molt, which was determined by the presence of indistinct dauer alae and an extra cuticle. The average ± SEM of at least three independent trials is shown. *n* = 68–155. L, larval stage; PD, postdauer; SDS, sodium dodecyl sulfate.

Like molting larvae, dauer larvae do not feed. We therefore tested the utility of fluorescent beads to identify dauer larvae. We isolated dauer larvae from starved plates by SDS selection and added them to bead-containing plates. Larvae were then monitored for the presence or absence of beads in the digestive tract. As expected, the dauer larvae failed to ingest any beads (Figure 3A). We continued to monitor dauer larvae as they recovered at 20°, and found that they began to display beads in their digestive tract after 2 hr, with nearly all larvae possessing beads after 180 min of recovery (Figure 3B). The ingestion of beads correlated well with the resumption of pumping and the loss of SDS resistance, both hallmarks of dauer recovery (Figure 3B), and this timing fits previously published data describing the process of dauer recovery (Cassada and Russell 1975). Thus, ingestion of beads is an early visible sign of dauer recovery.

### Using beads to identify dauer larvae from starved plates

Several methods can be employed to induce dauer formation in both small-scale and large-scale experiments. We tested the utility of the bead method to identify dauer larvae formed by three commonly used methods: starvation/crowding, exogenous pheromone, and mutations that cause constitutive dauer formation (“Daf-c” mutations).

First, we examined populations that had become crowded and had exhausted the food supply on the plate (“starved populations”). Allowing worms to starve is a simple method of generating large quantities of dauer larvae, but plates typically contain abundant nondauer stages as well. Thus, identifying the dauer larvae within the starved population is essential for further study. We washed wild-type worms off starved plates and added them to fresh bead-containing plates. After 30 min, we examined animals lacking beads and asked whether they possessed dauer characteristics including dauer alae and radial constriction (Cassada and Russell 1975). We found that essentially all worms lacking beads possessed these dauer characteristics (Figure 3C). Additionally, bead-lacking larvae were SDS resistant (Figure 3D), further indicating that these were dauer larvae (Cassada and Russell 1975). In contrast, worms with beads did not possess dauer alae or radial constriction (Figure 3C). Thus, a lack of beads is a reliable method to isolate dauer larvae from starved plates and can be used as an alternative to SDS resistance to recover dauer larvae from a starved population.

An SDS-independent method to isolate dauer larvae would be particularly useful when studying SDS-sensitive mutants, such as collagen or cuticulin mutants, or other mutants with abnormal cuticles. We asked whether a lack of beads could identify dauer larvae using two mutant strains with abnormal cuticles that result in a high degree of SDS sensitivity. We used a strain with a defective cuticulin, CB1 *dpy-1*, and a heterochronic mutant with a morphologically abnormal cuticle, VT1207 *lin-4*; *mir-48mir-241*; *lin-14mir-84*. Heterochronic mutants show defects in dauer morphology that may be due to aberrant seam cell fate (Liu and Ambros 1989). This particular mutant displays a reiteration of L2 seam cell fate, lack of adult alae when reproductively mature, and SDS-sensitivity (Karp and Ambros 2012).

We allowed CB1 and VT1207 strains to starve at 20°. Starved worms were washed off plates and added to fresh bead-containing plates for 30 min as described above. Similar to wild-type larvae, the lack of beads reliably identified larvae displaying dauer alae and radial constriction (Figure 3C*)*. In contrast, SDS treatment would have killed many of these larvae (Figure 3D). Therefore, lack of beads is a more effective indicator of dauer larvae than SDS resistance for experiments involving SDS-sensitive dauer larvae.

After determining that this method works to stage worms in both the continuous and dauer life histories, we tested variations on our method to determine its adaptability to different uses. The first variable we tested was time. We asked whether dauer larvae can be reliably identified by lack of beads when allowed to eat for fewer than 30 min. Beads were found in only 20% (5/25) of developing larvae 5 min after addition to the bead + OP50 mixture. At 15 min, almost all (18/19) developing larvae had ingested beads, and by 20 min of eating, beads were observed in 100% (41/41) of developing larvae. Therefore, depending on the degree of reliability needed for the particular assay, 15 or 20 min is sufficient to distinguish dauer and developing larvae based on beads. Next, we tested whether fluorescent objects other than the beads previously tested could be utilized. These include: 1) a GFP-expressing OP50 bacterial strain, and 2) fluorescent beads of a different color and size. As shown in Figure S4, GFP-OP50 can be used instead of beads but is much harder to visualize. The other beads tested were barely visible using a compound microscope and are not recommended (Figure S4).

### Using beads to identify dauer larvae formed by exogenous pheromone

A second method to stimulate dauer formation is to add dauer pheromone, comprised of a mix of ascaroside molecules (Jeong *et al.* 2005; Butcher *et al.* 2007), to the growth medium. Crude dauer pheromone extracted from growth medium varies from preparation to preparation and is often not 100% effective at inducing dauer formation, such that both continuously developing and dauer larvae are present on a plate. Pheromone is not only a useful tool to induce dauer formation but is itself a topic of investigation. For example, recent studies have examined the ability of different ascaroside mixtures to impact dauer formation and/or the responsiveness of different *C. elegans* isolates to particular ascarosides or pheromone preparations (Hollister *et al.* 2013; Diaz *et al.* 2014). For any of these analyses, a simple method to distinguish and quantify dauer and nondauer stages would be valuable.

We tested whether the absence of beads identifies dauer larvae formed by the addition of crude dauer pheromone to the growth medium. Populations of wild-type larvae were synchronized by allowing gravid adult hermaphrodites to lay eggs for 2 hr and then removing them. Embryos were laid on bead-containing plates supplemented with a crude preparation of dauer pheromone. The resulting larvae developing in the presence of dauer pheromone were monitored for the presence of beads and pumping. Beads were observed throughout the digestive tract in developing larvae. As in continuous development, molting larvae lacked beads (Figure 3E). As previously described (Golden and Riddle 1984), we found the L2d-dauer molt to be dramatically extended compared to other molts. The percentage of larvae pumping and feeding decreased beginning at 31 hr after egg laying (AEL), with only about 10% of larvae still eating and pumping by 34 hr AEL. By 50 hr AEL, two clear populations were present: a small population of nondauer larvae containing beads, and dauer larvae or larvae late within the L2d-dauer molt that lacked beads. Dauer alae, radial constriction, and SDS resistance are all acquired within the L2d-dauer molt, in that order (Golden and Riddle 1984). At 50 hr AEL, all bead-lacking larvae exhibited radial constriction and dauer alae, and most bead-lacking larvae were SDS-resistant (Figure 3F). In contrast, the ∼5–10% of larvae that exhibited beads were at the L4 stage. Therefore, these larvae represent the population that did not undergo dauer formation and, thus, the beads correctly separated the dauer and continuous populations.

### Using beads to identify dauer larvae formed by dauer-constitutive mutants

A third commonly used method to induce dauer formation takes advantage of dauer formation-constitutive (Daf-c) mutations that induce dauer formation at 24–25°, but permit reproductive growth at 15° or 20°. In principle, beads would be especially advantageous for studying these mutants because some also have dauer morphogenesis defects (see below), and they often display asynchronous exit from dauer.

**Figure 4:**
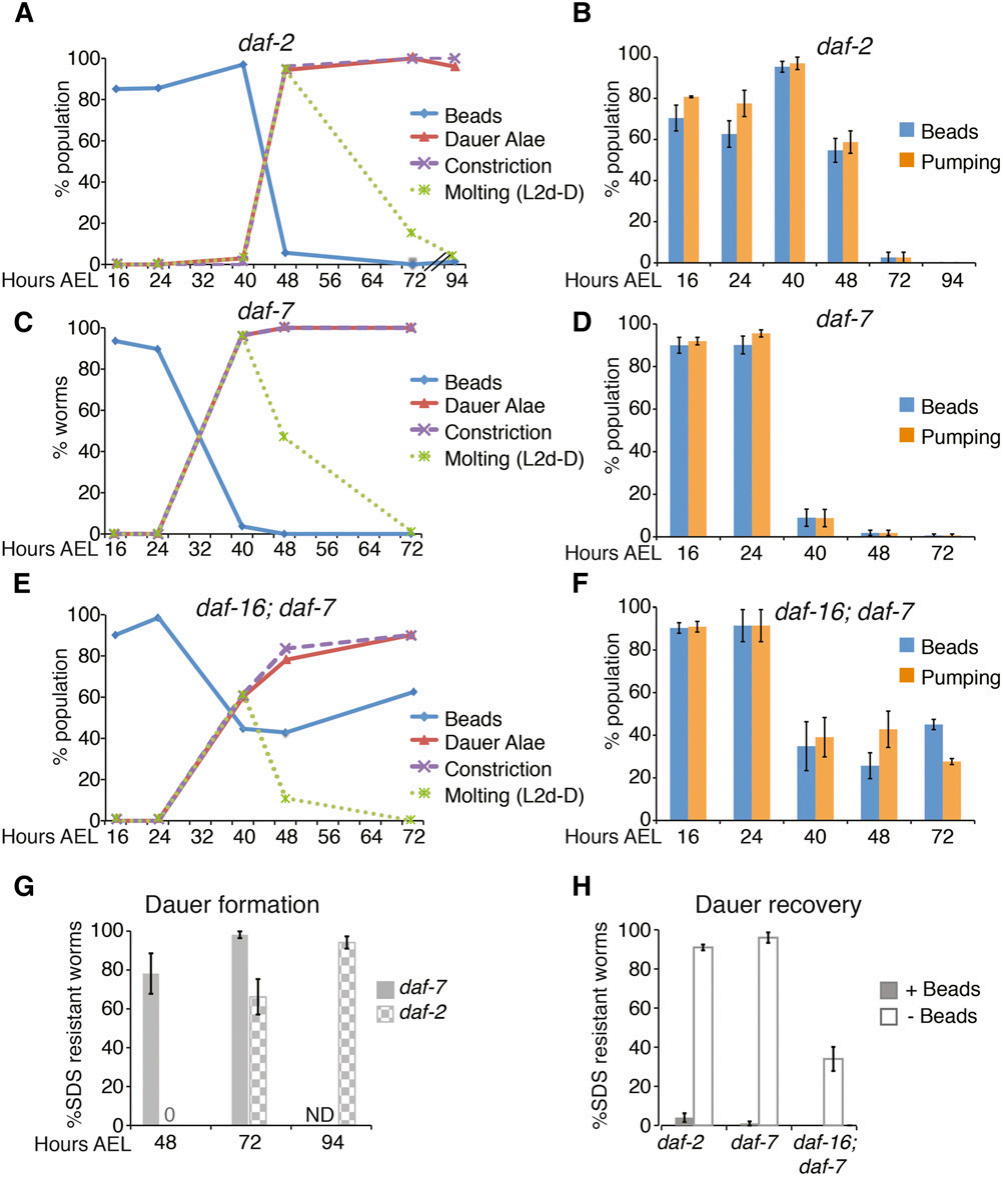
Lack of beads identifies dauer larvae and dauer-like larvae formed by Daf-c mutations. (A–F) Semisynchronous populations of Daf-c larvae were grown at 24° to induce dauer formation and monitored for the indicated characteristics. (A, C, and E) At least three independent trials were performed and the combined data are shown here. The average and SEM are shown in Table S2. Data were collected on a compound microscope. (B, D, and F) At least three independent trials were performed and the average ± SEM is shown here. Data were collected on a dissecting microscope. (A) *daf-2(e1370)*. Note that *daf-2* larvae grow more slowly than the other strains. (*n* > 45). (B) *daf-2(e1370)* (*n* > 65). (C) *daf-7(e1372)* (*n* > 45). (D) *daf-7(e1372)* (*n* > 100). (E) *daf-16(mgDf50)*; *daf-7(e1372)* (*n* > 45). (F) *daf-16(mgDf50)*; *daf-7(e1372)* (*n* > 70). (G) Bead-lacking larvae were tested for SDS resistance (*n* > 50). SDS resistance is acquired late in the L2d-dauer molt. At 48 hr AEL, SDS resistance is high in *daf-7* larvae, which have completed or nearly completed molting into dauer. In contrast, *daf-2* does not begin to acquire SDS resistance until 72 hr AEL. (H) Bead-lacking dauer larvae were transferred to new bead-containing plates and shifted to lower temperature (20°) to stimulate recovery. Approximately half of the population displayed beads in their digestive tract 1 d later. Bead-containing and bead-lacking larvae were tested for SDS resistance (*n* > 150). AEL, after egg laying; Daf-c, dauer constitutive; L, larval stage; SDS, sodium dodecyl sulfate.

We examined two Daf-c mutants that are often used as tools to induce dauer formation: *daf-7(e1372)*, encoding a mutant version of a dauer-opposing TGFβ signal (Ren *et al.* 1996), and *daf-2(e1370)*, encoding a mutant version of an insulin/insulin-like growth factor receptor (Kimura *et al.* 1997). In both cases, the results were similar to the wild-type in that dauer larvae and molting larvae failed to ingest beads, whereas other stages displayed beads in their digestive tract (Figure 4, A–D and Table S2). A lack of beads correlated with dauer characteristics including dauer alae, radial constriction, lack of pumping, and SDS resistance (Figure 4, A–D and G). We note that *daf-2(e1370)* developed more slowly than wild-type or *daf-7(e1372)*, a phenotype that has been observed before in continuously developing *daf-2* larvae (Ruaud *et al.* 2011).

One difficulty with using *daf-7(e1372)* and *daf-2(e1370)* mutations for some applications is that dauer recovery occurs relatively asynchronously with respect to wild-type (Vowels and Thomas 1992; Thomas *et al.* 1993; Malone *et al.* 1996). Thus, if recovering dauer or postdauer larvae are to be studied, these can be difficult to distinguish from larvae within the dauer stage. SDS resistance cannot be utilized because SDS-treatment kills the recovering dauer larvae. We find the presence of beads to be a reliable indicator of dauer recovery in *daf-7(e1372)* and *daf-2(e1370)* mutant larvae. *daf-7* or *daf-2* dauer larvae were shifted to temperatures conducive to recovery on bead-containing plates. The next day, approximately half of the population displayed beads in their digestive tract. Bead-containing larvae are SDS-sensitive, whereas larvae without beads are SDS-resistant (Figure 4H).

### Using beads to identify partial dauer larvae

There are many interesting mutants in the dauer formation pathways that are defective in certain aspects of dauer morphogenesis, such that in the dauer stage they lack particular dauer characteristics. These mutants, called “partial dauer” or “dauer-like,” include both Daf-c and Daf-d (dauer defective) alleles (Albert and Riddle 1988; Vowels and Thomas 1992; Gottlieb and Ruvkun 1994; Antebi *et al.* 1998). The Daf-d strains typically do not enter dauer in response to starvation conditions, but many can be driven into dauer by combining the Daf-d allele with a Daf-c allele in a parallel genetic pathway (Vowels and Thomas 1992; Larsen *et al.* 1995). Beads may be particularly useful to identify dauer-like larvae in these mutant backgrounds because they do not display some hallmarks of dauer formation that are used to identify dauer larvae, for example SDS resistance (see next paragraph).

To test the utility of beads in identifying dauer-like larvae, we focused on a *daf-16(mgDf50)*; *daf-7(e1372)* strain. *daf-16* encodes a FOXO transcription factor that acts downstream of insulin signaling to promote dauer formation (Ogg *et al.* 1997; Lin *et al.* 1997). *daf-16(mgDf50)*, a null mutant, is Daf-d (Ogg *et al.* 1997). *daf-7(e1372)* is a Daf-c mutation that can induce dauer formation in the *daf-16(0)* background (Vowels and Thomas 1992; Larsen *et al.* 1995; Ogg *et al.* 1997). *daf-16*; *daf-7* dauer-like larvae possess only some dauer characteristics: they secrete dauer alae and display radial constriction of the body, but not of the pharynx. They do not accumulate substantial fat stores, and pumping does not fully cease during dauer (Vowels and Thomas 1992; Ogg *et al.* 1997). Finally, *daf-16*; *daf-7* dauer-like larvae are only partially SDS-resistant: they generally survive a 10 min SDS treatment, but not a 40 min SDS treatment (Figure S5). Since 10 min is sufficient to kill wild-type larvae of all stages, even if they possess a dauer cuticle (Figure S5), this 10 min SDS treatment, together with dauer alae, offer the most reliable current methods to distinguish *daf-16* dauer-like larvae from other stages.

The ability to identify *daf-16*; *daf-7* dauer-like larvae is essential to their study, as growth at the dauer-inducing temperature (24°–25°) results in a mixed dauer-like and nondauer population. Nondauer larvae arise from two sources. Some *daf-16*; *daf-7* larvae grown at dauer-inducing temperatures bypass dauer formation altogether and develop continuously (Larsen *et al.* 1995; Narasimhan *et al.* 2011). Other *daf-16*; *daf-7* larvae do enter dauer, but then exit spontaneously (Gottlieb and Ruvkun 1994) (Figure S6). Study of these mutants is valuable because of the role that *daf-16* plays in regulating cell fate and quiescence during dauer (Narbonne and Roy 2006; Karp and Greenwald 2013). A simple, SDS-independent approach to identify *daf-16*; *daf-7* dauer-like larvae would facilitate this type of study, and is likely to be applicable to other dauer-like mutants.

To test whether a lack of beads is a reliable indicator of the dauer stage in *daf-16*; *daf-7* dauer-like larvae, we grew a semisynchronous population of *daf-16*; *daf-7* larvae at 24° on bead-containing plates. From 16–72 hr after egg laying, *daf-16*; *daf-7* larvae were monitored for dauer characteristics including SDS resistance, dauer alae, and radial constriction of the body (Figure 4, E and F). Any *daf-16*; *daf-7* larvae that bypassed dauer formation were removed from the plates as L4 larvae or young adults after scoring. Similar to *daf-7* larvae, the presence of beads in *daf-16*; *daf-7* larvae greatly diminished at 40 hr AEL, as larvae are molting into dauer (Figure 4, E and F and Table S2). Unlike *daf-7* larvae, the population of *daf-16*; *daf-7* larvae never fully lacked beads or pumping (Figure 4, E and F and Table S2).

**Figure 5:**
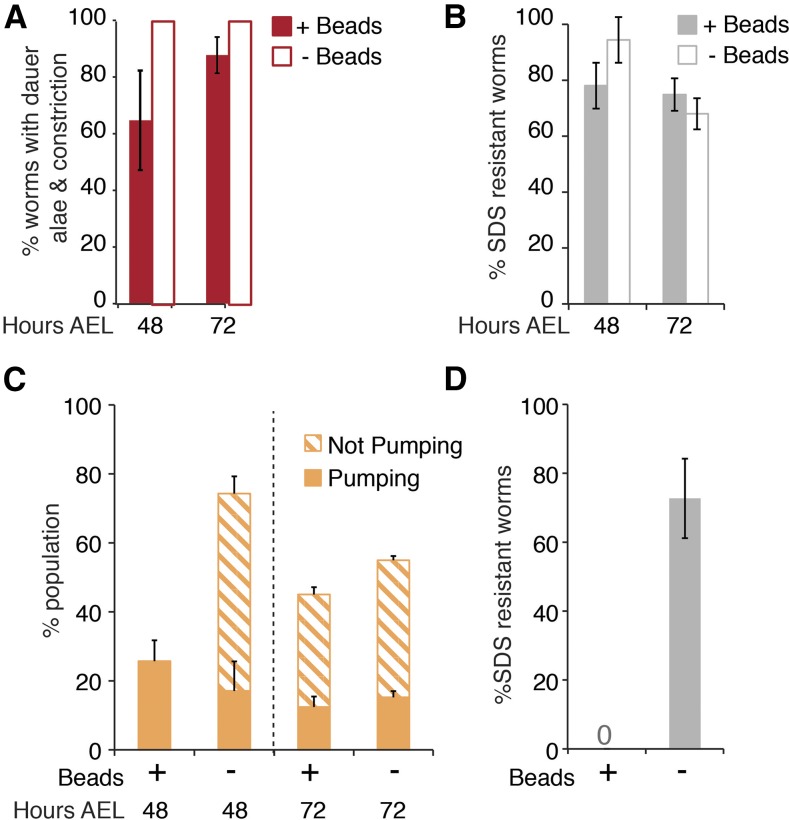
Dauer characteristics of bead-containing and bead-lacking *daf-16*; *daf-7* dauer-like larvae. (A–C) Semisynchronous populations of *daf-16*; *daf-7* larvae were grown at 24° on bead-containing plates. At 48 and 72 hr AEL, bead-containing and bead-lacking larvae were tested for the indicated characteristics. The average ± SEM of at least three independent trials is shown. (A) Beads, dauer alae, and radial constriction were assessed on a compound microscope. Dauer alae and radial constriction correlated perfectly. (*n* = 47–91). (B) 10 min SDS resistance assays were performed, and viability was assessed on a dissecting microscope. (*n* = 70–142). (C) Beads and pumping were assayed on a dissecting microscope. At 48 hr, AEL all bead-containing larvae were pumping. However, bead-lacking larvae comprised a mixture of pumping and nonpumping animals. At 72 hr AEL, both bead-containing and bead-lacking populations included both pumping and nonpumping animals. Thus, beads and pumping do not correlate in *daf-16*; *daf-7* dauer-like larvae, unlike control strains and *daf-16*; *daf-7* predauer stages. (*n* = 74–100). (D) Semisynchronous populations of *daf-16(mgDf50)*; *daf-7(e1372)* larvae were grown on bead-lacking plates at 24° for 71.5 hr. Larvae were then transferred to bead-containing plates and allowed to feed for 30 min. Bead-containing larvae (+Beads; 12% of the total population) and bead-lacking larvae (−Beads; 88% of the total population) were then assayed for SDS resistance. The average ± SEM of three independent trials is shown. (*n* = 65). AEL, after egg laying; SDS, sodium dodecyl sulfate.

To determine whether the bead-containing population of *daf-16*; *daf-7* larvae is different from the bead-lacking population at 48 and 72 hr AEL, we examined these populations of larvae for SDS resistance, dauer alae, and radial constriction. We found that 100% of bead-lacking larvae displayed dauer alae and radial constriction at both timepoints (Figure 5A). Furthermore, at 48 hr AEL, 94% of bead-lacking larvae were SDS resistant, though this declined to 68% at 72 hr AEL (Figure 5B). Therefore, nearly all bead-lacking *daf-16*; *daf-7* larvae are within the dauer stage, particularly at 48 hr AEL.

Surprisingly, bead-containing *daf-16*; *daf-7* larvae also displayed a high penetrance of these dauer characteristics (60–90%; Figure 5, A and B). Therefore, unlike the other strains examined, many bead-containing *daf-16*; *daf-7* larvae appear to be within the dauer stage. These dauer-like larvae may possess beads because they continue to feed. Alternatively, *daf-16*; *daf-7* dauer-like larvae may fail to empty their digestive tract prior to molting into dauer. To distinguish between these two possibilities, we grew *daf-16*; *daf-7* and control *daf-7* larvae at 24° on bead-lacking plates until 72 hr AEL. We then SDS-selected dauer and dauer-like larvae and added them to plates containing beads. Because roughly half the population of *daf-16*; *daf-7* larvae at 72 hr AEL contain beads (Figure 4, E and F), and because SDS resistance is equally high among bead-containing and bead-lacking *daf-16*; *daf-7* larvae (Figure 5B), we expected that approximately half of the SDS-resistant *daf-16*; *daf-7* larvae would have beads in their digestive tract if they had been grown on bead-containing plates. If *daf-16*; *daf-7* larvae feed, we would expect larvae to begin ingesting beads within 20 min, as 100% of wild-type nondauer larvae contain beads after 20 min of exposure to bead-containing food (see above). After 45–60 min on bead-containing plates, 0/74 *daf-7* and 0/66 *daf-16*; *daf-7* larvae contained beads in their digestive tract. The lack of beads in *daf-16*; *daf-7* dauer-like larvae indicates that they do not feed.

Consistent with the notion that the presence of beads in *daf-16*; *daf-7* larvae found on bead-containing plates is not due to feeding, we find that the tight correlation between beads and pumping was abolished in *daf-16*; *daf-7* larvae at 48 and 72 hr AEL, the times at which dauer-like larvae had formed (Figure 5C). This uncoupling of beads and pumping was never observed in wild-type, *daf-2*, or *daf-7* larvae at any stage examined. We therefore conclude that some *daf-16*; *daf-7* larvae do not empty their digestive tract prior to the L2d-dauer molt.

We next took advantage of the finding that *daf-16*; *daf-7* dauer-like larvae do not feed, in order to test whether feeding at 72 hr AEL could distinguish larvae within the dauer stage from larvae that had either bypassed dauer or were beginning to recover. We grew *daf-16*; *daf-7* larvae on bead-lacking plates at 24° until 71.5 hr AEL. Larvae were then transferred to bead-containing plates where they were incubated for 30 min. After incubation, bead-containing larvae and bead-lacking larvae were tested for SDS resistance. As expected, most of the larvae lacked beads, and this bead-lacking population displayed a high penetrance (72%) of SDS resistance (Figure 5D). However, a small bead-containing population was observed and these larvae were all SDS-sensitive, suggesting that they were no longer in the dauer stage (Figure 5D). Therefore, this method can be used to distinguish dauer-like larvae from recovering dauer-like larvae and will be useful for the characterization of mutants that form partial dauers.

The ability to positively identify recovering *daf-16*; *daf-7* dauer-like larvae prompted us to test whether the presence of beads could be used for the study of postdauer larvae, particularly prior to the PDL3 molt when they still possess dauer alae. Like *daf-7* mutants, *daf-16*; *daf-7* dauer-like larvae recover asynchronously. In *daf-7* mutants, identification of individual PDL3 larvae is possible without beads by looking for resumption of development, for example the division of the vulval precursor cells (Euling and Ambros 1996; Karp and Greenwald 2013). However, this criterion cannot distinguish PDL3 in *daf-16*; *daf-7* larvae, which aberrantly display such divisions during dauer (Karp and Greenwald 2013).

Therefore, we tested whether beads could be used to identify postdauer *daf-16*; *daf-7* larvae. We grew *daf-16*; *daf-7* larvae on bead-containing plates at 24° to induce dauer formation. We then picked bead-lacking dauer-like larvae to fresh bead-containing plates and shifted them to the permissive temperature for recovery. When approximately half of the population had beads in their digestive tract, larvae were tested for SDS sensitivity. All of the bead-containing larvae were SDS sensitive, confirming that they had recovered from dauer (Figure 4H). Therefore, the presence of beads can be used to identify recovered/postdauer *daf-16*; *daf-7* larvae.

We note, however, that only 34% of larvae lacking beads were SDS-resistant (Figure 4H). Thus, lack of beads after stimulation to recover is not a reliable indicator of dauer larvae that have not yet initiated recovery or have failed to recover. Nevertheless, the ability to positively identify postdauer *daf-16*; *daf-7* larvae provides a tool that makes it possible to study a stage that was previously inaccessible in these mutants.

## Discussion

In this work we have validated the use of fluorescent beads to aid in staging *C. elegans* larvae during development. When worms are fed beads mixed with the bacterial food source, the lack of beads in the digestive tract indicates an empty gut, a situation we observe in both dauer larvae and developing larvae in the process of molting. Based on the context, as well as other aspects of morphology, these two populations can be distinguished. We did not observe molting larvae on plates that had been starved for at least several days, indicating that molting larvae are quite rare in these populations. Therefore, lack of beads is a reliable indicator of the dauer larva stage rather than molting larvae when examining starved plates.

Fluorescent beads offer a new tool to distinguish molting from nonmolting larvae. Expression of *mlt-10*::*gfp-pest* has been used to mark larvae within the molt (*e.g.*, Gissendanner and Kelley 2013; MacNeil *et al.* 2013). A recently described method using bioluminescence from a luciferase-GFP expressing transgene can mark the intermolt period (Olmedo *et al.* 2015). In contrast, the use of beads does not require a transgene and, thus, the method can be applied to any existing strain. Given that the expression of thousands of genes oscillates with the molting cycle (Kim *et al.* 2013), and that many mutant strains develop somewhat asynchronously, a simple method to identify larvae between molts will be valuable for many studies, including the quantification of gene expression levels during development.

Fluorescent beads will also enhance the ability to screen for either dauer or postdauer larvae. Beads are easy to add to the food source, and individual candidate mutants can be directly visualized. For example, genetic screens using beads may enable the identification of novel SDS-sensitive dauer formation mutants that shed light on the regulation of metabolism and/or aging, *e.g.*, *daf-31* (Chen *et al.* 2014). In addition, the use of fluorescent beads may be combined with a COPAS Biosort (wormsorter) to enable the large-scale isolation and quantification of dauer, postdauer, or molting larvae. Finally, a simple method to identify dauer larvae may be valuable for experiments using dauer formation mutants, even when the focus is not on dauer *per se*. For example, dauer-constitutive mutants in the insulin signaling pathway are used for aging research. Beads may therefore facilitate a wide range of *C. elegans* genetic research.

## Supplementary Material

Supplemental Material
