# Supplementary material for: Fluorescent Beads Are a Versatile Tool for Staging Caenorhabditis elegans in Different Life Histories
Source: G3 (Bethesda). 2016 Apr 29;6(7):1923–33. doi: 10.1534/g3.116.030163 (PMC4938646; doi:10.1534/g3.116.030163)
Supplement: Supplemental Material [file supp_6_7_1923__index.html]

Fluorescent Beads Are a Versatile Tool for Staging Caenorhabditis elegans in Different Life Histories — Supplemental Material 

# Fluorescent Beads Are a Versatile Tool for Staging *Caenorhabditis elegans* in Different Life Histories

## Supplemental Material for Nika *et al.*, 2016

**Files in this Data Supplement:**

- Figure S1 - Correlation between beads and pumping during the L1 molt in individual larvae. (.pdf, 304 KB)
- Figure S2 - Correlation between beads and pumping in individual larvae. (.pdf, 325 KB)
- Figure S3 - *mlt-10::GFP-pest* expression. (.pdf, 250 KB)
- Figure S4 - Comparison of different fluorescent objects. (.pdf, 301 KB)
- Figure S5 - Ten-minute SDS resistance assays are sufficient to identify dauer larvae. (.pdf, 240 KB)
- Figure S6 - *daf-16; daf-7* dauer-like larvae spontaneously recover at non-permissive temperatures. (.pdf, 249 KB)
- Table S1 - Timing of removal of beads from the digestive tract by defecation. (.xlsx, 46 KB)
- Table S2 - Dauer constitutive mutants during dauer formation. (.xlsx, 48 KB)
