# Supplementary material for: Fluorescent Beads Are a Versatile Tool for Staging Caenorhabditis elegans in Different Life Histories
Source: G3 (Bethesda). 2016 Apr 29;6(7):1923–33. doi: 10.1534/g3.116.030163 (PMC4938646; doi:10.1534/g3.116.030163)
Supplement: Supplemental Material [file supp_g3.116.030163_FigureS1.pdf]

Time	Worm 1		Worm 2		Worm 3		Worm 4		Worm 5	
(min.)	Beads	Pumping	Beads	Pumping	Beads	Pumping	Beads	Pumping	Beads	Pumping
-50	+++	+++							+++	+++
-45	+++	+++							+++	+++
-40	+++	+++							+++	+++
-35	+++	+++							+++	+++
-30	+++	+++			+++	+++			+++	+++
-25	+++	+++			+++	+++			+++	+++
-20	+++	+++	+++	+++	+++	+++			+++	+++
-15	+	+++	+++	+++	+++	+++			+++	+++
-10	+	+++	+++	+	+++	+++	+	+++	+++	+++
-5	+	+	+	+	+++	+++	+	+++	+	+
0	-	+	-	-	-	+	-	+	-	+
5	-	-	-	-	-	+	-	+	-	-
10	-	-	-	-	-	-	-	-	-	-
15	-	-	-	-	-	-	-	-	-	-
20	-	-	-	-	-	-	-	-	-	-
25	-	-	-	-	-	-	-	-	-	-
30	-	-	-	-	-	-	-	-	-	-
35	-	-	-	-	-	-	-	-	-	-
40	-	-	-	-	-	-	-	-	-	-
45	-	+/-	-	-	-	-	-	-	-	-
50	-	-	-	-	-	-	-	-	-	-
55	-	-	-	-	-	-	-	-	-	-
60	-	-	-	-	-	+/-	-	-	-	-
65	-	-	-	-	-	-	-	-	-	-
70	-	-	-	-	-	-	-	-	-	-
75	-	-	-	-	-	-	-	-	-	-
80	-	-	-	-	-	+	-	-	-	-
85	-	-	-	-	-	+	-	-	-	+
90	-	-	-	-	-	+++	-	+	+	+++
95	-	-	-	-	+	+++	+	+++	+++	+++
100	-	+	-	+	+++	+++	+++	+++	+++	+++
105	-	+	-	+++	+++	+++	+++	+++	+++	+++
110	+	+++	+	+++	+++	+++	+++	+++		
115	+++	+++	+	+++	+++	+++	+++	+++		
120	+++	+++	+++	+++	+++	+++	+++	+++		
125	+++	+++	+++	+++	+++	+++	+++	+++		
130			+++	+++	+++	+++	+++	+++		
135			+++	+++	+++	+++	+++	+++		
140			+++	+++	+++	+++	+++	+++		
145			+++	+++	+++	+++	+++	+++		
150			+++	+++			+++	+++		

Figure S1. Correlation between beads and pumping during the L1 molt in individual larvae. Individual N2 larvae were incubated at 24˚C on bead-containing plates and monitored on the dissecting microscope. These data are from L1 larvae observed in parallel in one of five independent trials (see Figures 2, S2). Time 0 (in minutes) is defined as the time when beads were completely expunged from the digestive tract. *Beads*: +++ many beads throughout the digestive tract, + few beads or beads visible only in the posterior of the larva, - beads absent from the digestive tract. *Pumping*: +++ normal pharyngeal pumping rate, + slow pumping, +/- one pump within 5 minutes, - no pumping.
