# Supplementary material for: Fluorescent Beads Are a Versatile Tool for Staging Caenorhabditis elegans in Different Life Histories
Source: G3 (Bethesda). 2016 Apr 29;6(7):1923–33. doi: 10.1534/g3.116.030163 (PMC4938646; doi:10.1534/g3.116.030163)
Supplement: Supplemental Material [file supp_g3.116.030163_FigureS2.pdf]

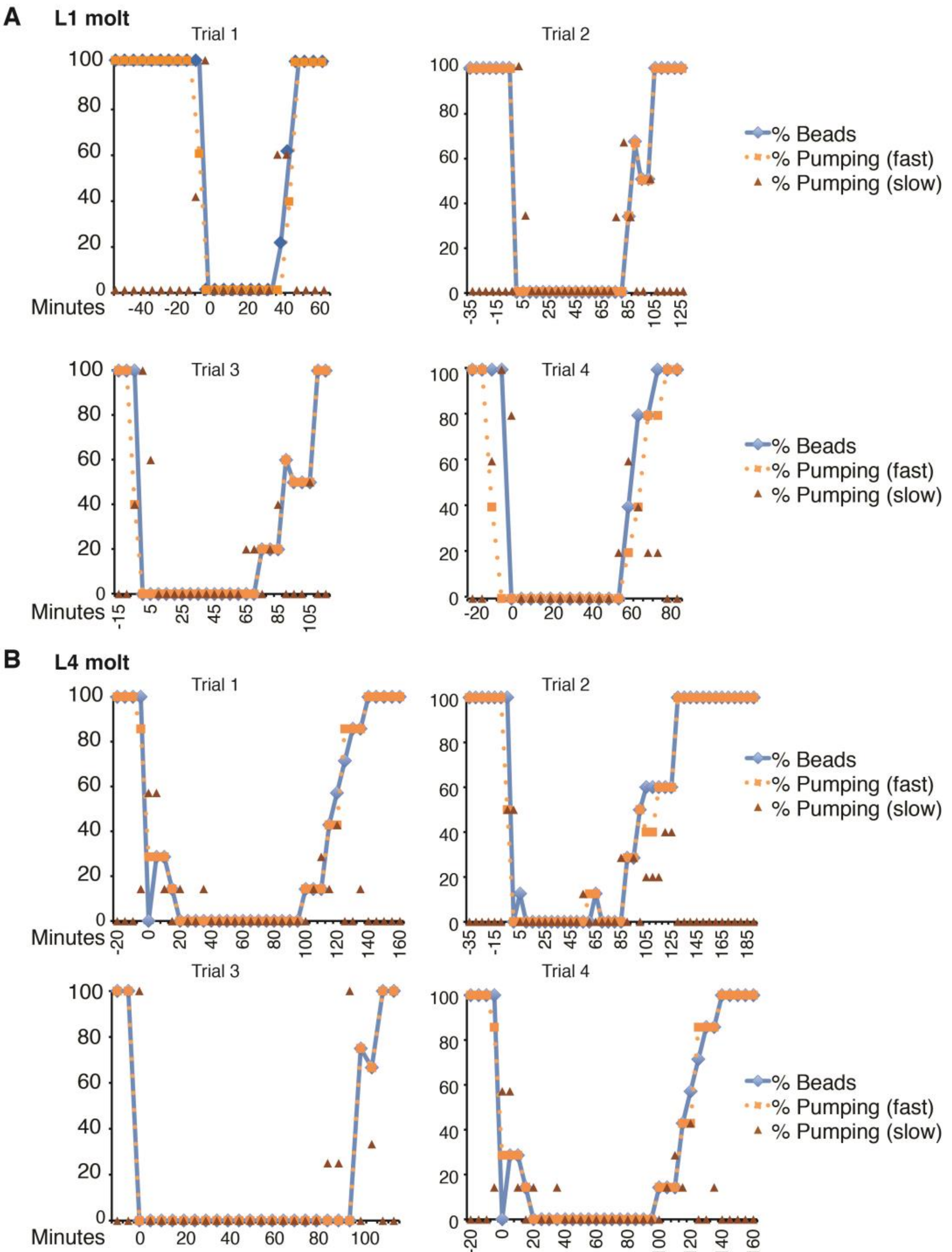

Figure S2. Correlation between beads and pumping in individual larvae. Individual N2 larvae grown on bead-containing plates at 24˚C were monitored on a dissecting microscope for beads and pumping every five minutes throughout the L1 or L4 molt. Each independent trial consisted of 3-8 larvae and the average data for each trial is shown here. An additional trial from the L1 molt is shown in Figure 2B and Figure S1. Data are from a total of 23 L1 larvae and 23 L4 larvae. Although the precise length of the molt varies from trial to trial and from individual to individual, in all cases beads and pumping are tightly correlated. However, slow pumping can occur in the presence or absence of beads at the beginning and end of the molting period. Time 0 is defined as the time (minutes) that beads are completely expunged from the gut.
