# Supplementary material for: Fluorescent Beads Are a Versatile Tool for Staging Caenorhabditis elegans in Different Life Histories
Source: G3 (Bethesda). 2016 Apr 29;6(7):1923–33. doi: 10.1534/g3.116.030163 (PMC4938646; doi:10.1534/g3.116.030163)
Supplement: Supplemental Material [file supp_g3.116.030163_FigureS3.pdf]

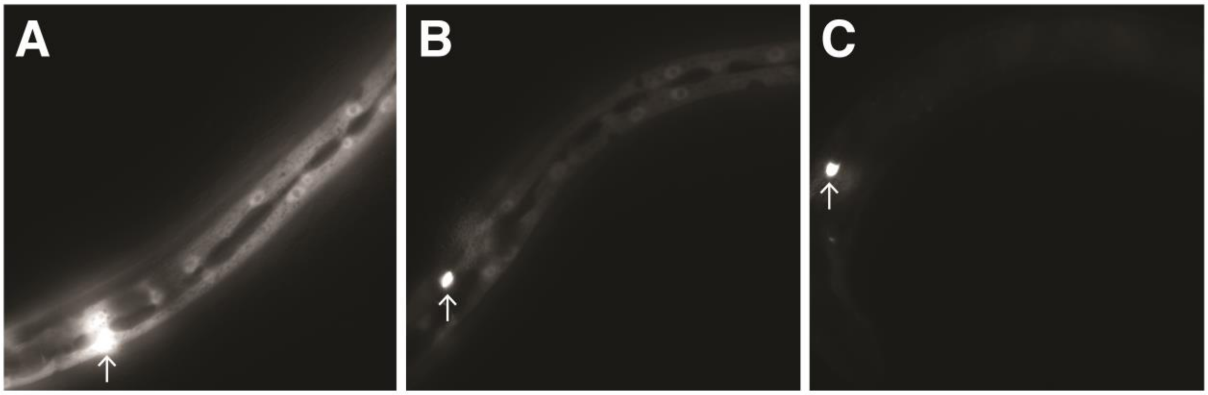

D

	+ Beads		- Beads	
Hours	GFP	Pumping	GFP	Pumping
10	0/80	80/80	0	0
10.5	0/78	78/78	0	0
11	0/79	79/79	0	0
11.5	0/76	76/76	4/4	0/4
12	0/81	81/81	41/41	1/41
12.5	0/40	40/40	41/41	2/41
13	0/45	45/45	42/42	1/42
13.5	0/40	40/40	31/31	0/31
14	0/61	61/61	11/11	0/11
14.5	0/78	78/78	2/2	1/2

Figure S3. *mlt-10::GFP-pest* expression. Representative images of *mgIs49[mlt-10::GFP-pest]* expression were taken at the same exposure time (15ms) and at 400X magnification [40X objective] on a compound microscope. (A) Strong GFP expression. (B) Faint GFP expression. (C) No GFP expression. Arrows indicate *ttx-3p::GFP*, an injection marker in this strain. (D) Data from Figure 2C are broken down to show that the bead-containing larvae fail to strongly express GFP whereas bead-lacking larvae do strongly express GFP. “+Beads” = bead-containing larvae; “- Beads” = bead-lacking larvae; “Hours” = Hours post-hatching; “GFP” = strong GFP expression. Data from at least 3 independent trials are aggregated here. Note that faint GFP expression does occur during the intermolt period when larvae are pumping.
