# Supplementary material for: Fluorescent Beads Are a Versatile Tool for Staging Caenorhabditis elegans in Different Life Histories
Source: G3 (Bethesda). 2016 Apr 29;6(7):1923–33. doi: 10.1534/g3.116.030163 (PMC4938646; doi:10.1534/g3.116.030163)
Supplement: Supplemental Material [file supp_g3.116.030163_FigureS4.pdf]

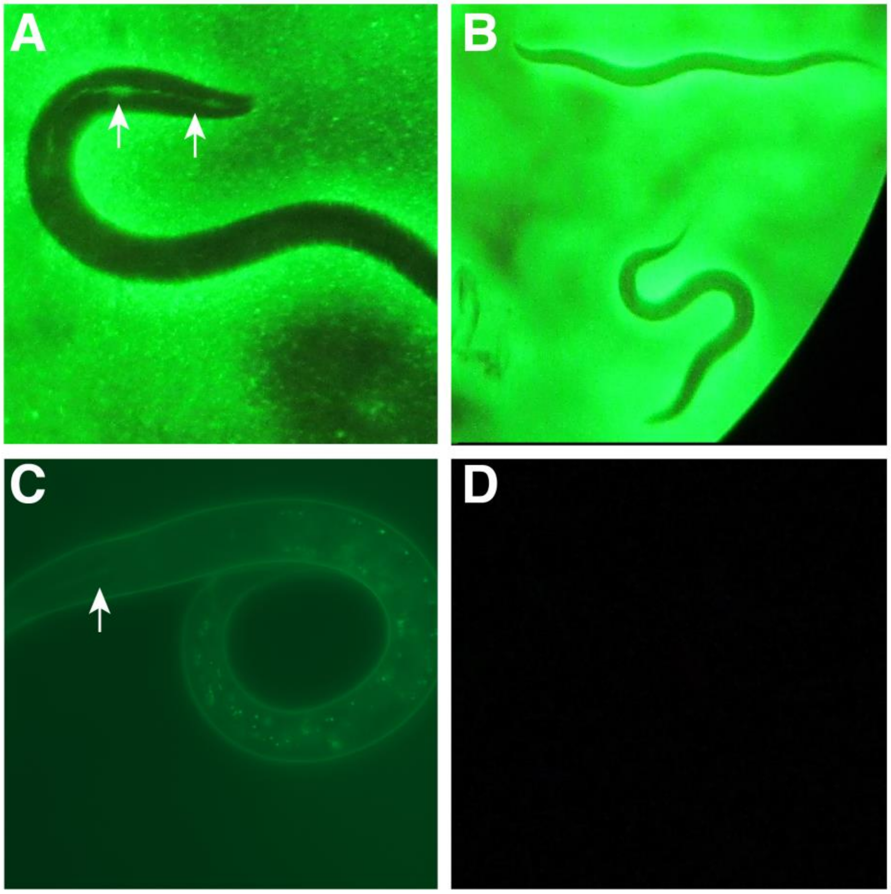

Figure S4. Comparison of different fluorescent objects. Asynchronous starved populations were washed off of standard NGM + OP50 plates and added to plates containing fluorescent beads or GFP-expressing OP50. Animals were incubated at 24˚C for 30 minutes before visualizing. Arrows indicate visible fluorescent beads or bacteria in the digestive tract. (A, D) 300X magnification [using a 10X objective] on a dissecting microscope. (B) 100X magnification [using a 1X objective] on a dissecting microscope. (C) 400X magnification [40X objective] on a compound microscope. (A-B) GFP-OP50 is more difficult to see than beads. At higher magnification (A), GFP is seen in the pharynx but not in the rest of the digestive tract. At lower magnification (B) it is impossible to distinguish feeding and non-feeding worms. (C-D) Yellow-green 0.03μm fluorescent beads (Sigma L5155) were barely visible even on the compound microscope (C) and were not visible using the dissecting microscope (D).
