# Supplementary material for: Fluorescent Beads Are a Versatile Tool for Staging Caenorhabditis elegans in Different Life Histories
Source: G3 (Bethesda). 2016 Apr 29;6(7):1923–33. doi: 10.1534/g3.116.030163 (PMC4938646; doi:10.1534/g3.116.030163)
Supplement: Supplemental Material [file supp_g3.116.030163_FigureS5.pdf]

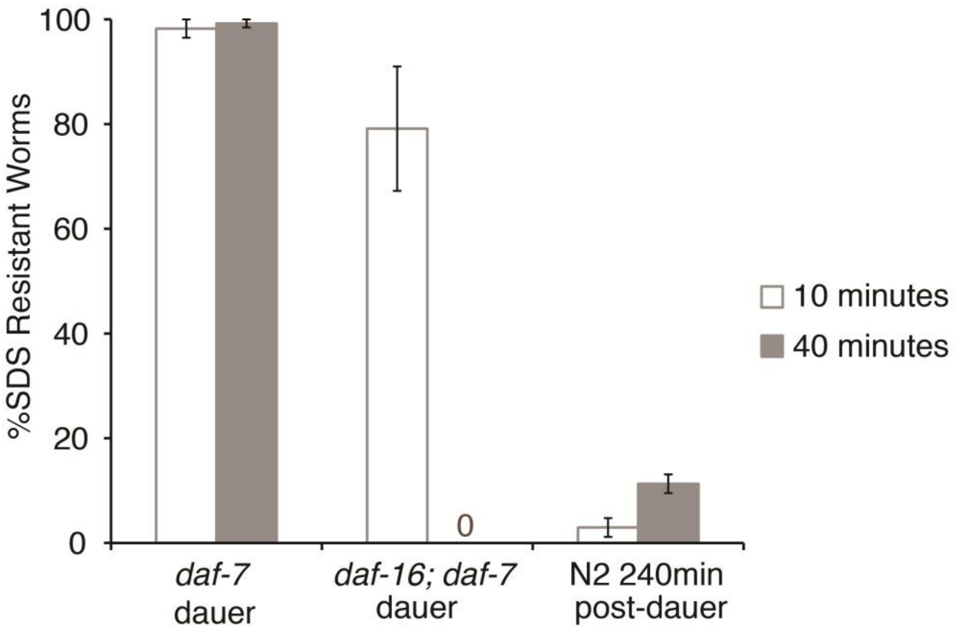

Figure S5. Ten-minute SDS resistance assays are sufficient to identify dauer larvae. Dauer or post-dauer larvae were incubated in 1% SDS for either 10 minutes or 40 minutes prior to washing and plating. *daf-7* dauer larvae survived either treatment, whereas *daf-16; daf-7* dauer-like larvae survived only the 10-minute treatment, demonstrating partial SDS-sensitivity in this strain. Ten minutes is sufficient to kill non-dauer larvae, even when such larvae possess a dauer cuticle. N2 dauer larvae were first selected by a 40 minute SDS treatment, and then allowed to recover for 240 minutes. At this time, larvae are eating and pumping (see Figure 3B), but have not yet molted out of their dauer cuticle. These larvae are sensitive to SDS, including both 10-minute and 40-minute treatments. The average of at least 3 independent trials +/- SEM is shown. $n > 50$.
