# Supplementary material for: Fluorescent Beads Are a Versatile Tool for Staging Caenorhabditis elegans in Different Life Histories
Source: G3 (Bethesda). 2016 Apr 29;6(7):1923–33. doi: 10.1534/g3.116.030163 (PMC4938646; doi:10.1534/g3.116.030163)
Supplement: Supplemental Material [file supp_g3.116.030163_FigureS6.pdf]

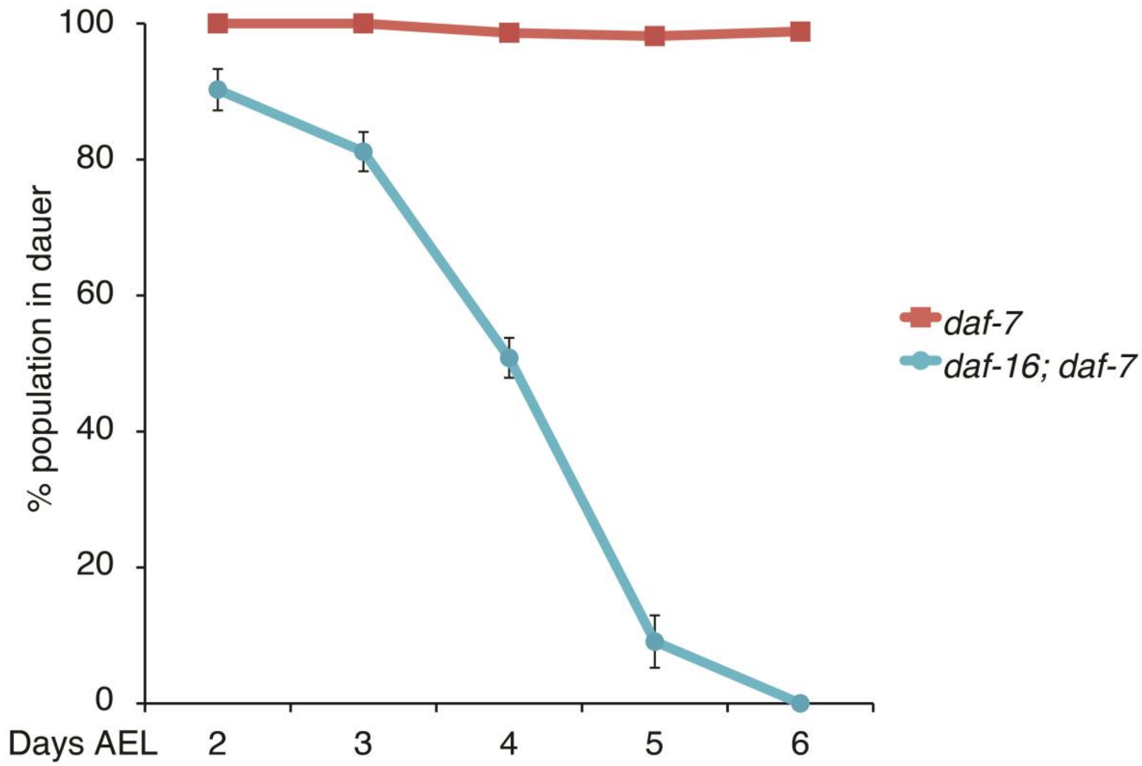

Figure S6. *daf-16; daf-7* dauer-like larvae spontaneously recover at non-permissive temperatures. Synchronous populations of *daf-7* and *daf-16; daf-7* embryos were incubated in parallel at 24˚C to induce dauer formation. Two days after egg-laying (AEL), most larvae had entered dauer, with a small number of *daf-16; daf-7* larvae that bypassed dauer formation. Worms were maintained at 24˚C and each day, plates were inspected and any larvae that had recovered to L4 were counted and removed. The L4 stage was determined by body size and morphology. Worms that retained dauer morphology were counted as dauer larvae. By six days AEL, 100% of *daf-16; daf-7* dauer larvae had recovered, while nearly all *daf-7* larvae remained in dauer. This experiment was repeated for a total of four independent trials. The average of the four trials, +/- SEM, is shown. Note that for *daf-7*, the error bars are too small to be visible. n > 200.
